# Disparities in Stage at Diagnosis among Hispanic Patients with Gastric Cancer in the United States

**DOI:** 10.3390/cancers16193308

**Published:** 2024-09-27

**Authors:** Antoine Jeri-Yabar, Liliana Vittini-Hernandez, Renzo Aller-Rojas, Francisco Arias-Reyes, Sirish Dharmapuri

**Affiliations:** 1 Department of Internal Medicine, Icahn School of Medicine at Mount Sinai Morningside/West, New York, NY 10023, USA; 2 Department of Internal Medicine, University of Texas Rio Grande Valley, McAllen, TX 78503, USAfrancisco.reyes.al@gmail.com (F.A.-R.); 3 Department of Hematology/Oncology, Icahn School of Medicine at Mount Sinai West, New York, NY 10023, USA; siirish.dharmapuri@gmail.com

**Keywords:** cancer disparities, gastric cancer, disparities, Hispanic

## Abstract

**Simple Summary:**

This study investigates racial disparities in the stage of gastric cancer at diagnosis and overall survival among different racial groups using data from the SEER database (2018–2021). The retrospective cohort study of 18,984 patients found that Hispanics are 19% more likely to be diagnosed at a later stage of gastric cancer compared to non-Hispanics. Additionally, both Hispanics and Black patients showed poorer overall survival compared to Non-Hispanic Whites. The disparities are attributed to factors such as healthcare access, insurance coverage, language barriers, and preventive health service utilization. These findings highlight the need for targeted interventions to address these disparities in cancer outcomes.

**Abstract:**

Introduction: Racial disparities in gastric cancer outcomes, including stage at diagnosis and overall survival, continue to affect Hispanic and non-Hispanic populations. This study aims to evaluate these disparities across different racial groups. Patients and methods: We conducted a retrospective cohort study using SEER data from 2018 to 2021, including 18,984 patients diagnosed with gastric cancer. Patients were selected based on ICD-O-3 codes for “stomach” with malignant behavior. Using ordered logistic regression, the association between race and stage at diagnosis was analyzed, while Cox proportional hazards models were used to assess OS and CSS. Results: Hispanic patients were significantly more likely to be diagnosed at a later stage compared to non-Hispanic patients (OR: 1.19; 95% CI: 1.10–1.28). Both Hispanic and Black patients had worse OS compared to Non-Hispanic Whites (HR 1.10 CI 1.03–1.17, *p* = 0.003 and HR 1.13 CI 1.04–1.22, *p* = 0.002, respectively) as well as CSS. Conclusions: Hispanic patients are more likely to be diagnosed with advanced-stage gastric cancer and have poorer survival outcomes compared to non-Hispanic Whites. These disparities may be linked to differences in healthcare access, insurance, language barriers, and preventive care utilization.

## 1. Introduction

Racial disparities in cancer outcomes are a critical issue in the United States, reflecting underlying social and systemic inequalities [1]. While disparities across various cancer types have been well-documented, gastric cancer remains an area of particular concern, especially among Hispanic/Latinx populations [1,2,3,4,5,6]. Data from the 2020 U.S CENSUS revealed that Hispanic/Latinxs constitute approximately 19.1% of the total U.S population [7]. Within this demographic group, significant racial and ethnic disparities in disease burden and associated mortality have been observed, particularly in gastric cancer [8].

The American Cancer Society reports that between 2015 and 2019, Hispanic/Latinx individuals experienced nearly double the incidence rates and mortality rates for stomach cancer than their White counterparts [8]. Moreover, a recent study from the University of Texas Health Science Center (UTHSC) found that Hispanic patients diagnosed with cardiac gastric adenocarcinoma (GCA) were more likely to present with late-stage GCA compared to non-Hispanic White patients [9]. Although gastric cancer mortality has decreased across populations in the US, disparities in gastric cancer mortality among racial–ethnic groups persist [10].

To the best of our knowledge, no studies have been conducted studying the disparities in stage at diagnosis of gastric cancer among Hispanics in the Surveillance, Epidemiology, and End Results (SEER) database [11]. The aim of the study is to investigate and elucidate the disparities in the stage at diagnosis of gastric cancer among Hispanic populations in the United States compared to other racial and ethnic groups and perform a survival analysis to evaluate overall survival and cause-specific survival among races.

## 2. Methods

We analyzed the Surveillance, Epidemiology, and End Results (SEER) incidence database that was submitted on November 2022 and issued in April 2023 [11], which included data from 2018 to 2021. Selection of the population studied was based on the ICD-O-3 2023 revision expanded primary site and morphology: “stomach” and site and morphology behavior recode for analysis: “malignant”, and microscopically confirmed. ICD-O-3 Sites included were C160–C169, specific to stomach. We excluded patients with incomplete data on our independent variables: sex, age, race (Hispanic, Non-Hispanic Black, Non-Hispanic White, Non-Hispanic Asian/Pacific Islander, Non-Hispanic American Indian), staging (according to extent of disease 2018+ on SEER) months from diagnosis to treatment, household income (individual level), chemotherapy, radiation therapy, survival months, vital status, marital status, rural/urban area of living. Only patients 19 or older were included. Patients with missing data on key variables such as race, tumor, stage, and survival were excluded from the analysis. Of the 19,923 patients initially identified, 939 (4.8%) were excluded due to missing data, resulting in the final sample size of 18,984.

### 2.1. Outcomes

The primary outcome of the study was to analyze the stage of gastric cancer (categorized broadly stage I, II, III, IV) at diagnosis according to different races and sociodemographic variables. The secondary outcomes were to measure overall survival, measured from the date of diagnosis to the date of death from any cause, with patients censored at the last known date they were alive and cause-specific survival defined as net survival measure representing cancer survival in the absence of other causes of death.

### 2.2. Statistical Analysis

A descriptive analysis was performed with frequencies and percentages for categorical variables and continuous variables with median and IQR due to skewed distribution of date to describe the study population’s demographic and clinical characteristics stratified by race/ethnicity. Ordered logistic regression was used to study the association between the race and the stage of gastric cancer at diagnosis. The models estimated odds ratios (ORs) and 95% confidence intervals with stage of diagnosis treated as an ordinal outcome. The ordered logistic regression model assumes proportional odds, meaning that the relationship between the independent variables and the outcome is consistent across the different thresholds of the dependent variable. To verify this assumption, we conducted separate binary logistic regressions for each possible cutoff of the stage variable and compared the coefficients, and it was found that the proportional odds assumption holds in our data. The secondary outcome, overall survival, was defined as the time of diagnosis to death from any cause, and patients who were alive were censored at the time of last recording. Overall survival (OS) was estimated with the Kaplan–Meier product limit method and compared using the log-rank test. A multivariate Cox model was used to assess differences among race, while adjusting for age, sex, rural/urban area, marital status, stage at diagnosis, chemotherapy, radiation, and months from diagnosis to treatment. Cause-specific survival (CSS) was defined as the time from diagnosis to death from gastric cancer and patients who died of other causes were censored. All statistical tests were two sided, with a *p* value of <0.05 considered statistically significant. Analyses were conducted using STATA v.17.0 [12].

### 2.3. Ethics

As this study was based on the Surveillance, Epidemiology, and End Results database, no ethics approval was needed as this is a public database with national data.

## 3. Results

After applying the inclusion and exclusion criteria on the SEER database, 18,984 patients with gastric cancer were included in the study. Of the population included in the study, 20.99% were Hispanic (H), 50.07% were Non-Hispanic White (NHW), 14.31% were Non-Hispanic Asian/Pacific Islander (NHA), 13.72% were Non-Hispanic Black (NHB), and 0.91% were Non-Hispanic American Indian (NHAI). Most patients (42.74%) were in the 65–79 years age category. There was a statistically significant difference in age distribution across racial groups (*p* < 0.001). H patients exhibited a higher incidence of gastric cancer compared to other racial groups within the 20–49 years age category (24.92%; *p* < 0.001), followed by NHAI patients (16.76%; *p* < 0.001). Males constituted 59.53% of the population, while females constituted 40.47% in total and in every race subgroup, with a significant difference in gender distribution across all racial groups (*p* < 0.001). Socioeconomic status was measured by household income, with 72.29% of the population included in the study reporting a household income of >$75,000 (*p* < 0.001). Most of the population lived in metropolitan areas (90.45%) (*p* < 0.001), with a minority living in non-metropolitan areas (9.55%). Most patients were married (63.54%) (*p* < 0.001). Regarding stage at diagnosis, most individuals were diagnosed at stage IV (43.64%), while 27.77% were diagnosed at stage I (*p* < 0.001). In the analysis of racial subgroups, 48.41% of H patients were diagnosed at stage IV, while 23.51% were diagnosed at stage I. Among NHW patients, 43.43% were diagnosed at stage IV and 28.91% were diagnosed at stage I. For NHB patients, 41.90% were diagnosed at stage IV and 30.88% were diagnosed at stage I. For NHA patients, 38.79% were diagnosed at stage IV and 27.20% were diagnosed at stage I. Additionally, among NHAI patients, 47.40% were diagnosed at stage IV and 24.86% were diagnosed at stage I (*p* < 0.001). Regarding studied treatment, chemotherapy was given to 64.53% of the population and 22.44% were given radiotherapy. The median survival was 10 months for H patients, 11 months for NHW patients, 11 months for NHB patients, 12 months for NHA patients, and 9 months for NHAI patients (*p* < 0.001). These results are shown in Table 1.

The ordered logistic regression analysis exploring factors associated with stage of diagnosis in gastric cancer can be found in Table 2. Hispanics are significantly more likely to be diagnosed at a later stage compared to the reference group (NHWs), with both unadjusted and adjusted (OR = 1.19 CI 1.10–1.28) models showing statistical significance (*p* < 0.001). NHB, NHA, and NHAI groups did not show significant differences in the stage of diagnosis in the adjusted model. Regarding age, all age subgroups were more likely to be diagnosed at earlier stages when comparing to the reference group (20–49 years). The older the age group, the less likely they were to be diagnosed at later stages (50–64 years, OR: 0.83 CI 0.75–0.90, *p* < 0.001; 65–79 years, OR: 0.69 CI 0.63–0.76, *p* < 0.001; >80 years, OR 0.66 CI 0.59–0.74, *p* < 0.001). Females had a lower odds ratio of being diagnosed at a later stage (OR = 0.87 CI 0.82–0.92; *p* < 0.001) than males. Household income did not show a significant association with the stage of diagnosis in the unadjusted analysis and were excluded from the adjusted model due to high multicollinearity. Living in a metropolitan area was not associated with being diagnosed at later stage and neither was marital status. As per tumor histology, cystic/mucinous/serous tumors had higher odds of being diagnosed at later stages as well as squamous cell tumors when compared to gastric adenocarcinoma (OR: 1.36; 1.25–1.49; *p* < 0.001) (OR: 1.96; 1.63–2.36; *p* < 0.001). Tumors with overlapping anatomical sites also had an increased risk of being found at later stages (OR 1.33; 1.21–1.46; *p* < 0.001).

**Figure 1:**
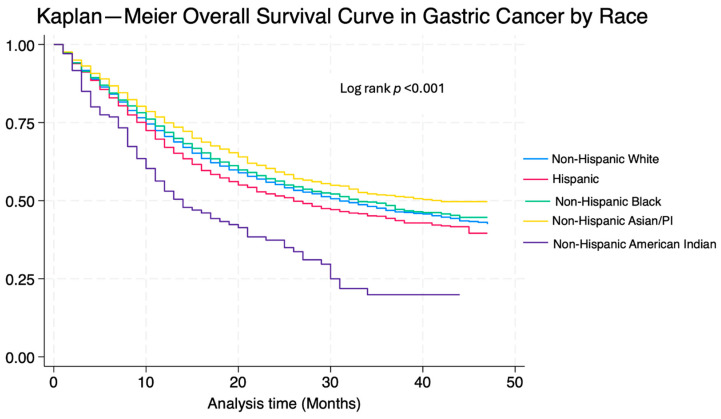
Kaplan–Meier overall survival curve in gastric cancer by race.

**Figure 2:**
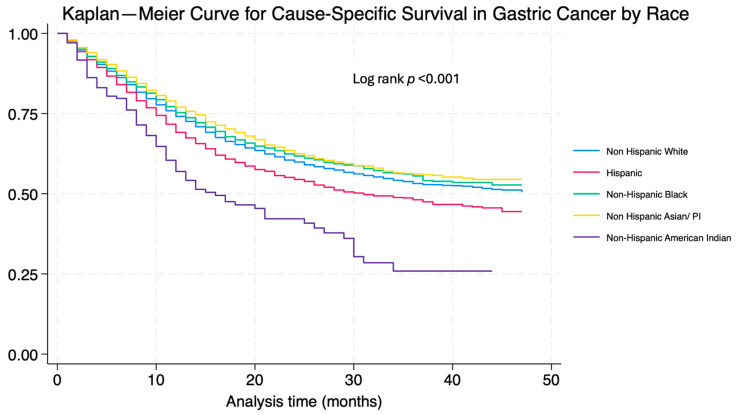
Kaplan–Meier cause-specific survival curve in gastric cancer by race.

The multivariate Cox proportional hazard regression analysis focusing on factors that affect overall survival among gastric cancer patients is shown in Table 3. H and NHB patients had an increased risk of mortality when compared to NHW patients (HR 1.10 CI 1.03–1.17, *p* = 0.003 and HR: 1.13; 1.04–1.22; *p* = 0.002, respectively). Regarding age, patients older than 80 years old had a higher risk of worse outcomes when compared to a younger population (HR 1.83 CI 1.66–2.01; *p* < 0.001). Being married was associated with better survival (HR 0.89 CI 0.83–0.95, *p* = 0.001). A higher stage at diagnosis was associated with worse survival (stage II HR 1.33, 1.19–1.49; *p* < 0.001; stage III 2.36, 2.13–2.62; *p* < 0.001; stage IV 5.44, 4.99–5.95; *p* < 0.001). Cause-specific survival showed an increase in mortality in H, B, and NHAI patients when compared to NHW patients (HR 1.15 CI 1.07–1.23, *p* < 0.001; HR 1.09 CI 1.09–1.19 *p* = 0.03; HR 1.01 CI 0.92–1.09 *p* = 0.813; HR 1.72 CI 1.38–2.15 *p* < 0.001, respectively); further information can be found in Table 4. Kaplan–Meier curves on OS and CSS can be found in Figure 1 and Figure 2, respectively.

## 4. Discussion

Our study revealed that Hispanics in the U.S. are 19% more likely to be diagnosed of gastric cancer at later stages when compared to Non-Hispanic Whites. This significant difference could be attributed to various factors, including disparities in healthcare access among these racial groups [13]. Notably, healthcare access among the Hispanic population in the U.S. shows significant disparities influenced by socioeconomic and systemic factors [14,15]. Multiple barriers to access exist, such as recent migration to the United States and unfamiliarity with the U.S. healthcare system, which impede individuals from obtaining appropriate and timely care [15]. Additionally, fear of stigma and deportation among undocumented individuals seeking care further exacerbates barriers [16]—for example, it has been shown that a lack of legal documentation is a significant barrier to cervical cancer screening among Hispanic women [17]. It has also been shown that a lack of citizenship and lower acculturation levels are associated with an increased risk of not following recommended cancer screenings in women’s healthcare [18].

Moreover, a study focusing on the clinical presentation patterns and survival outcomes of Hispanic patients with gastric cancer revealed that Hispanics were significantly less likely to have a primary care provider (PCP) when compared to non-Hispanic Whites (46% vs. 75%; *p* < 0.05) [19]. Patients lacking a PCP often presented with abdominal symptoms in emergency departments and were more likely to be diagnosed with metastatic disease [19]. Language barriers further exacerbate these disparities, with up to 30 to 40 percent of the over 50 million Latinos in the United States reporting less than proficient English skills [14]. Research has shown that language-concordant care can significantly improve outcomes in healthcare settings, suggesting that language barriers likely contribute to diagnostic and treatment delays in the Hispanic population [20,21].

Our analysis reveals an intriguing inverse association between age and the propensity for a later-stage diagnosis of gastric cancer, suggesting that older patients are more likely to undergo screening sooner. This observation raises critical questions about the diagnostic journey of younger patients, particularly those under 50, who may face delayed diagnoses. Notably, early-onset gastric cancer often presents at an advanced stage [22]. This finding underscores the necessity for considering gastric cancer in the differential diagnosis of younger patients <50 years presenting with non-specific gastric symptoms, as there is a rising incidence of early-onset gastric cancer [23]. Investigating the referral patterns of primary care physicians based on the age of patients presenting with similar gastric symptoms at different ages is essential. We also found that females presented a lower probability of being diagnosed at later stages when compared to males, which might reflect differences in health-seeking behavior, as women are more likely to seek cancer-related information [24] and tend to report their symptoms more accurately compared to men [25]. On the contrary, men tend to have a more reductionist approach to their healthcare needs and tend to ignore their symptoms [26], which could partly explain why they are diagnosed at a later stage.

Our study also showed that Hispanics and Non-Hispanic Black patients exhibit significantly worse overall survival and cause-specific survival compared to Non-Hispanic Whites, even after adjustments for other variables. This raises critical concerns about the underlying causes, such as potential disparities in access to care [15] and differences in treatment quality [6]. Additionally, potential biological differences in tumor aggressiveness or development have been reported before, which showed a higher frequency of a poor prognosis-associated molecular subtype in Hispanic/Latino populations [27]. Past studies have highlighted significant variations in treatment across different ethnicities regarding treatment across stages I-IV (all *p* < 0.001), with Asian/Pacific Islander patients having the highest rate of surgery plus radiation [28]. Notably, Williams et. al failed to find a statistically significant difference in survival or clinical features between Latinos and Non-White Latinos with gastric cancer [29]; however, the study population only included 193 patients, with 126 being Latinos. Furthermore, our study found no significant difference in survival or increased likelihood of early-stage diagnosis among Non-Hispanic Asians/Pacific Islanders, which contrasts with previous studies that have indicated Asians are more likely to be diagnosed at earlier stages and demonstrate better survival outcomes regardless of tumor location compared to the White population [30].

In order to improve disparities in survival outcomes for minority racial groups in gastric cancer and other cancers, several strategies are being implemented and should be prioritized. Studies have shown that minority populations, especially non-Hispanic Black and Hispanic groups, have low participation in clinical trials [31]. Approaches such as using patient navigators and translators are being employed to address this issue [31,32]. Furthermore, a systematic review indicated that highly segmented interventions are needed to improve cancer screening among various racial minorities [33]. Additionally, providing culturally tailored education and outreach programs is crucial for raising awareness about gastric cancer risks and the importance of early detection within minority communities, and subsequently, improving outcomes. These efforts can involve partnerships with community leaders and local organizations to build trust and effectively disseminate information [1]. Further research should focus on incidence disparities in minority populations in the U.S. to develop and implement preventive screening methods and address health disparities in the stage at diagnosis of this disease.

The SEER database provides a unique strength to our study by offering a large, population-based sample with extensive follow-up data, which enhances the generalizability of our findings to the broader U.S. population. The comprehensive nature of the SEER database allows for a detailed analysis across diverse racial and ethnic groups, which is particularly important for examining disparities in cancer outcomes. However, the SEER database also has several limitations that must be acknowledged. First, the database lacks detailed information on certain potential confounders, such as individual-level socioeconomic factors, comorbidities, and insurance coverage, which could influence survival outcomes. Additionally, SEER data are limited in capturing detailed treatment information beyond basic categorizations, which could obscure differences in treatment quality or adherence. Furthermore, the retrospective nature of the study limits our ability to infer causality from the observed associations. Lastly, another limitation is that our follow-up period was from 2018 to 2021 (3 years); this was due to the SEER database including staging since 2018, which was an important variable for our primary outcome.

Future research should aim to incorporate qualitative insights and patient-reported outcomes to better understand barriers to early diagnosis and develop more effective, culturally competent interventions. Additionally, there is a pressing need for culturally sensitive, multidisciplinary approaches to cancer care that address the unique needs of diverse patient populations to reduce disparities and improve stage at diagnosis and survival outcomes among all groups.

## 5. Conclusions

In conclusion, Hispanics are disproportionately diagnosed at later stages of gastric cancer when compared to Non-Hispanic Whites and have worse survival outcomes. These findings suggest systemic barriers and potential disparities in access to healthcare providers and indicate the pressing need for targeted interventions to improve early detection and equitable access to healthcare. Additionally, the inverse relationship between age and stage at diagnosis highlight the critical need for enhanced awareness among those under 50 years old. Future research should aim to uncover the root causes of these disparities to form strategies that ensure timely diagnosis within the Hispanic community.

## Figures and Tables

**Table 1:** Demographic data of patients with gastric cancer from SEER database 2018–2021.

Characteristics (*n* = 18,984)	Hispanic (*n* = 3985)	Non-Hispanic White (*n* = 9505)	Non-Hispanic Black (*n* = 2604)	Non-Hispanic Asian/Pacific Islander (*n* = 2717)	Non-Hispanic American Indian/Alaska Native (*n* = 173)	Total	*p* Value
Demographics							
Age							<0.001 †
20–49 years	993 (24.92%)	766 (8.06%)	356 (13.67%)	319 (11.74%)	29 (16.76%)	2463 (12.97%)	
50–64 years	1375 (34.50%)	2764 (29.08%)	931 (35.75%)	733 (26.98%)	52 (30.06%)	5855 (30.84%)	
65–79 years	1274 (31.97%)	4528 (47.64%)	1033 (39.67%)	1197 (44.06%)	81 (46.82%)	8113 (42.74%)	
>80 years	343 (8.61%)	1447 (15.22%)	284 (10.91%)	468 (17.22%)	11 (6.36%)	2553 (13.45%)	
Sex							<0.001 †
Male	2092 (52.50%)	6160 (64.81%)	1392 (53.46%)	1552 (57.12%)	105 (60.69%)	11,301 (59.53%)	
Female	1893 (47.50%)	3345 (35.19%)	1212 (46.54%)	1165 (42.88%)	68 (39.31%)	7683 (40.47%)	
Socioeconomic Status							
Household Income							<0.001 †
<$35,000	14 (0.35%)	134 (1.41%)	39 (1.50%)	0 (0%)	0 (0%)	187 (0.99%)	
$35,000–$49,000	45 (1.13%)	404 (4.25%)	290 (11.14%)	9 (0.33%)	15 (8.67%)	763 (4.02%)	
$50,000–$64,000	355 (8.91%)	1323 (13.92%)	497 (19.09%)	70 (2.58%)	55 (31.79%)	2300 (12.12%)	
$65,000–$74,000	358 (8.98%)	1136 (11.95%)	381 (14.63%)	122 (4.49%)	15 (8.67%)	2012 (10.60%)	
$75,000–99,999	2295 (57.59%)	4068 (42.80%)	1047 (40.21%)	1295 (47.66%)	78 (45.09%)	8783 (46.27%)	
>$100,000	918 (23.04%)	2440 (25.67%)	350 (13.44%)	1221 (44.94%)	10 (5.78%)	4939 (26.02%)	
Metropolitan/Non-Metropolitan Area							<0.001 †
Non-Metropolitan	142 (3.56%)	1288 (13.55%)	242 (9.29%)	49 (1.80%)	92 (53.18%)	1813 (9.55%)	
Metropolitan	3843 (96.44%)	8217 (86.45%)	2362 (90.71%)	2668 (98.20%)	81 (46.82)	17,171 (90.45%)	
Marital Status							<0.001 †
Single	847 (21.25%)	1187 (12.49%)	762 (29.26%)	301 (11.08%)	26 (15.03%)	3123 (16.45%)	
Married	2410 (70.48%)	6353 (66.84%)	1230 (47.24%)	1954 (71.92%)	116 (67.05%)	12,063 (63.54%)	
Divorced	420 (10.54%)	1002 (10.54%)	335 (12.86%)	192 (7.07%)	20 (11.56%)	1969 (19.37%)	
Widowed	308 (7.73%)	963 (10.13%)	277 (10.64%)	270 (9.94%)	11 (6.36%)	1829 (9.63%)	
Stage at Diagnosis							<0.001 †
Stage I (IA, IB)	937 (23.51%)	2748 (28.91%)	804 (30.88%)	739 (27.20%)	43 (24.86%)	5271 (27.77%)	
Stage II (IIA, IIB)	546 (13.70%)	1373 (14.45%)	362 (13.90%)	457 (16.82%)	24 (13.87%)	2762 (14.55%)	
Stage III (IIIA, IIIB, IIIC)	573 (14.38%)	1256 (13.21%)	347 (13.33%)	467 (17.19%)	24 (13.87%)	2667 (14.05%)	
Stage IV	1929 (48.41%)	4128 (43.43%)	1091 (41.90%)	1054 (38.79%)	82 (47.40%)	8284 (43.64%)	
Histology							<0.001 †
Adenocarcinoma	2781 (69.79%)	6836 (71.92%)	1599 (61.41%)	1852 (68.16%)	135 (78.03%)	13,203 (69.55%)	
Cystic, mucinous or serous	727 (18.24%)	946 (9.95%)	233 (8.95%)	394 (14.50%)	28 (16.18%)	2328 (12.26%)	
Squamous cell	80 (2.01%)	242 (2.55%)	85 (3.26%)	59 (2.17%)	5 (2.89%)	471 (2.48%)	
Others	397 (9.96%)	1481 (15.58%)	687 (26.38%)	412 (15.16%)	5 (2.89%)	2982 (15.71%)	
Site of Tumor							<0.001 †
Cardia	606 (15.21%)	4118 (43.32%)	350 (13.44%)	437 (16.08%)	29 (16.76)	5540 (29.18%)	
Fundus	199 (4.99%)	521 (5.48%)	182 (6.99%)	161 (5.93%)	12 (7.51%)	1076 (5.67%)	
Body	1229 (30.84%)	1914 (20.14%)	800 (30.72%)	804 (29.59%)	53 (30.64%)	4800 (25.28%)	
Antrum (including pylorus)	854 (21.43%)	1106 (11.64%)	601 (23.08%)	720 (26.50%)	34 (19.65%)	3314 (17.46%)	
Overlap	331 (8.31%)	468 (4.92%)	178 (6.84%)	213 (7.84%)	17 (9.83%)	1207 (6.36%)	
Undefined	766 (19.22%)	1378 (14.50%	493 (18.93%)	382 (14.06%)	27 (15.61%)	3046 (16.05%)	
Chemotherapy							<0.001 †
Yes	2759 (69.23%)	6048 (63.63%)	1562 (59.98%)	1760 (64.78%)	122 (70.52%)	12,251 (64.53%)	
No	1226 (30.77%)	3457 (36.37%)	1042 (40.02%)	957 (35.22%)	51 (29.48%)	6733 (35.47%)	
Radiation							<0.001 †
Yes	684 (17.16%)	2656 (27.94%)	405 (15.55%)	476 (17.52%)	39 (22.54%)	4260 (22.44%)	
No	3301 (82.84%)	6849 (72.06%)	2199 (84.45%)	2241 (82.48%)	134 (77.46%)	14,724 (77.56%)	
Vital Status							<0.001 †
Alive	2444 (61.33%)	5876 (61.82%)	1661 (63.79%)	1799 (66.21%)	75 (43.35%)	11,855 (62.45%)	
Dead	1541 (38.67%)	3629 (38.18%)	943 (36.21%)	918 (33.79%)	98 (56.65%)	7129 (37.55%)	
Months of Survival							<0.001 ‡
Median (IQR)	10 (19)	11 (21)	11 (19)	12 (20)	9 (15)		

‡ Kruskal Wallis. † Chi-Squared test. IQR: Interquartile range.

**Table 2:** Ordered logistic regression analysis of factors associated with stage of diagnosis in gastric cancer.

Variable	Unadjusted OR (95% CI)	*p* Value	Adjusted OR (95% CI)	*p* Value
Race				
Non-Hispanic White	Ref.			
Hispanic	1.26 (1.18–1.35)	<0.001	1.19 (1.10–1.28)	<0.001
Non-Hispanic Black	0.93 (0.85–1.07)	0.076	1.08 (0.99–1.18)	0.061
Non-Hispanic Asian/Pacific Islander	0.93 (0.86–1.00)	0.086	0.99 (0.91–1.08)	0.928
Non-Hispanic American Indian	1.19 (0.90–1.58)	0.202	1.11 (0.83–1.48)	0.461
Age				
20–49 years	Ref.			
50–64 years	0.80 (0.73–0.87)	<0.001	0.83 (0.75–0.90)	<0.001
65–79 years	0.65 (0.59–0.70)	<0.001	0.69 (0.63–0.76)	<0.001
>80 years	0.60 (0.54–0.67)	<0.001	0.66 (0.59–0.74)	<0.001
Sex				
Male	Ref.			
Female	0.84 (0.79–0.88)	<0.001	0.87 (0.82–0.92)	<0.001
Socioeconomic Status				
<$65,000	Ref.			
≥$65,000–$99,999	1.04 (0.97–1.12)	0.241	1.04 (0.96–1.13)	0.293
≥$100,000	1.07 (0.98–1.16)	0.087	1.09 (0.99–1.20)	0.071
Metropolitan/Non-Metropolitan Area				
Rural	Ref.			
Metropolitan	1.03 (0.94–1.12)	0.481	0.97 (0.87–1.08)	0.631
Marital Status				
Single	Ref.			
Married	0.88 (0.81–0.94)	0.001	0.94 (0.87–1.02)	0.166
Divorced	0.88 (0.79–0.97)	0.016	0.95 (0.85–1.05)	0.364
Widowed	0.76 (0.68–0.85)	<0.001	0.98 (0.87–1.10)	0.781
Histology				
Adenocarcinoma	Ref.			
Cystic, mucinous or serous	1.39 (1.28–1.51)	<0.001	1.36 (1.25–1.49)	<0.001
Squamous cell	2.04 (1.70–2.45)	<0.001	1.96 (1.63–2.36)	<0.001
Others	0.40 (0.37–0.44)	<0.001	0.40 (0.37–0.44)	<0.001
Site of Tumor				
Cardias	Ref.			
Fundus	0.58 (0.51–0.66)	<0.001	0.79 (0.69–0.90)	0.001
Body	0.60 (0.56–0.64)	<0.001	0.68 (0.63–0.74)	<0.001
Antrum (including pylorus)	0.68 (0.63–0.74)	<0.001	0.67 (0.62–0.73)	<0.001
Overlap	1.22 (1.09–1.37)	<0.001	1.21 (1.07–1.36)	0.002
Undefined	1.10 (1.01–1.20)	0.018	1.33 (1.21–1.46)	<0.001

OR: Odds ratio 95% CI: 95% confidence intervals. Ordered logistic regression; adjusted for age, sex, metropolitan/non-metropolitan city, marital status, income, site of tumor, and histology.

**Table 3:** Multivariate Cox proportional hazard regression analysis of factors affecting survival.

Variable	Unadjusted Hazard Ratio (95% CI)	*p* Value	Adjusted Hazard Ratio (95% CI)	*p* Value
Race				
Non-Hispanic White	Ref.			
Hispanic	1.10 (1.03–1.16)	0.002	1.10 (1.03–1.17)	0.003
Non-Hispanic Black	0.96 (0.89–1.03)	0.294	1.13 (1.04–1.22)	0.002
Non-Hispanic Asian/Pacific Islander	0.85 (0.79–0.91)	<0.001	0.96 (0.89–1.04)	0.371
Non-Hispanic American Indian	1.79 (1.46–2.18)	<0.001	1.69 (1.37–2.07)	<0.001
Age				
20–49 years	Ref.			
50–64 years	0.93 (0.86–1.01)	0.089	1.01 (0.93–1.10)	0.698
65–79 years	0.98 (0.91–1.06)	0.739	1.16 (1.07–1.25)	<0.001
>80 years	1.42 (1.30–1.56)	<0.001	1.83 (1.66–2.01)	<0.001
Sex				
Male	Ref.			
Female	0.80 (0.76–0.84)	<0.001	0.85 (0.81–0.90)	<0.001
Socioeconomic Status (Household Income)				
<$65,000	Ref.			
≥$65,000-$99,999	0.93 (0.87–1.00)	0.05	0.96 (0.89–1.03)	0.334
≥$100,000	0.89 (0.82–0.96)	0.002	0.94 (0.86–1.02)	0.191
Metropolitan/Non-Metropolitan Area				
Rural	Ref.			
Metropolitan	0.86 (0.80–0.93)	<0.001	0.93 (0.85–1.02)	0.17
Marital Status				
Single	Ref.			
Married	0.89 (0.83–0.95)	0.001	0.89 (0.83–0.95)	0.001
Divorced	1.00 (0.91–1.10)	0.941	1.03 (0.94–1.14)	0.421
Widowed	1.16 (1.06–1.27)	0.001	1.09 (0.98–1.20)	0.079
Stage at Diagnosis				
Stage I	Ref.			
Stage II (IIA, IIB)	1.75 (1.57–1.95)	<0.001	1.33 (1.19–1.49)	<0.001
Stage III (IIIA, IIIB, IIIC)	3.13 (2.84–3.45)	<0.001	2.36 (2.13–2.62)	<0.001
Stage IV	6.33 (5.84–6.86)	<0.001	5.44 (4.99–5.95)	<0.001
Chemotherapy				
No	Ref.			
Yes	2.31 (2.18–2.45)	<0.001	1.16 (1.08–1.24)	<0.001
Radiation				
No	Ref.			
Yes	1.61 (1.53–1.69)	<0.001	1.27 (1.20–1.35)	<0.001
Site				
Cardias	Ref.			
Fundus	0.63 (0.56–0.70)	<0.001	1.10 (0.97–1.24)	0.122
Body	0.61 (0.57–0.65)	<0.001	0.91 (0.84–0.98)	0.021
Antrum (including pylorus)	0.80 (0.74–0.85)	<0.001	0.96 (0.89–1.04)	0.364
Overlap	1.15 (1.04–1.26)	0.003	1.17 (1.06–1.29)	0.001
Undefined	0.85 (0.79–0.91)	<0.001	1.14 (1.05–1.23)	0.001
Histology				
Adenocarcinoma	Ref.			
Cystic, mucinous or serous	1.35 (1.27–1.44)	<0.001	1.36 (1.27–1.46)	<0.001
Squamous cell	1.80 (1.59–2.04)	<0.001	1.43 (1.26–1.63)	<0.001
Others	0.14 (0.12–0.16)	<0.001	0.15 (0.13–0.18)	<0.001

HR: Hazard ratios; 95% CI: 95% confidence intervals. Cox regression model; adjusted for age, sex, metropolitan/non-metropolitan city, marital status, income, site of tumor, and histology.

**Table 4:** Multivariate Cox proportional hazard regression analysis of factors affecting cause-specific survival.

Variable	Unadjusted Hazard Ratio (95% CI)	*p* Value	Adjusted Hazard Ratio (95% CI)	*p* Value
Race				
Non-Hispanic White	Ref.			
Hispanic	1.18 (1.11–1.26)	<0.001	1.15 (1.07–1.23)	<0.001
Non-Hispanic Black	0.94 (0.86–1.02)	0.142	1.09 (1.01–1.19)	0.039
Non-Hispanic Asian/Pacific Islander	0.89 (0.82–0.96)	0.005	1.01 (0.92–1.09)	0.813
Non-Hispanic American Indian	1.82 (1.46–2.26)	<0.001	1.72 (1.38–2.15)	<0.001
Age				
20–49 years	Ref.			
50–64 years	0.88 (0.81–0.95)	0.003	0.98 (0.90–1.07)	0.804
65–79 years	0.86 (0.80–0.93)	<0.001	1.07 (0.99–1.17)	0.081
>80 years	1.19 (1.08–1.31)	<0.001	1.68 (1.51–1.86)	<0.001
Sex				
Male	Ref.			
Female	0.80 (0.76–0.85)	<0.001	0.87 (0.82–0.92)	<0.001
Socioeconomic Status (Household Income)				
<$65,000	Ref.			
≥$65,000-$99,999	0.91 (0.70–1.18)	0.494	0.80 (0.61–1.03)	0.094
≥$100,000	0.77 (0.61–0.97)	0.031	0.70 (0.55–0.91)	0.008
Metropolitan/Non-Metropolitan Area				
Rural	Ref.			
Metropolitan	0.88 (0.81–0.96)	0.004	0.97 (0.88–1.08)	0.652
Marital Status				
Single	Ref.			
Married	0.87 (0.81–0.93)	0.001	0.88 (0.82–0.95)	0.001
Divorced	0.98 (0.89–1.08)	0.763	1.04 (0.94–1.14)	0.436
Widowed	1.04 (0.94–1.15)	0.403	1.04 (0.94–1.17)	0.383
Stage at Diagnosis				
Stage I	Ref.			
Stage II (IIA, IIB)	2.24 (1.97–2.54)	<0.001	1.62 (1.42–1.85)	<0.001
Stage III (IIIA, IIIB, IIIC)	4.34 (3.87–4.87)	<0.001	3.04 (2.69–3.43)	<0.001
Stage IV	9.06 (8.21–10.01)	<0.001	7.30 (6.57–8.12)	<0.001
Chemotherapy				
No	Ref.			
Yes	2.81 (2.63–3.01)	<0.001	1.28 (1.19–1.39)	<0.001
Radiation				
No	Ref.			
Yes	1.61 (1.53–1.71)	<0.001	1.25 (1.18–1.33)	<0.001
Site				
Cardias	Ref.			
Fundus	0.58 (0.51–0.66)	<0.001	1.06 (0.93–1.21)	0.359
Body	0.60 (0.55–0.64)	<0.001	0.91 (0.84–0.98)	0.036
Antrum (including pylorus)	0.78 (0.73–0.84)	<0.001	0.94 (0.87–1.03)	0.207
Overlap	1.23 (1.12–1.35)	0.003	1.22 (1.10–1.35)	0.001
Undefined	0.85 (0.78–0.91)	<0.001	1.14 (1.04–1.25)	0.001
Histology				
Adenocarcinoma	Ref.			
Cystic, mucinous or serous	1.44 (1.35–1.54)	<0.001	1.39 (1.30–1.50)	<0.001
Squamous cell	1.88 (1.65–2.14)	<0.001	1.47 (1.29–1.68)	<0.001
Others	0.07 (0.06–0.09)	<0.001	0.08 (0.07–0.10)	<0.001

HR: Hazard ratios; 95% CI: 95% confidence intervals. Cox regression model; adjusted for age, sex, metropolitan/non-metropolitan city, marital status, income, site of tumor, and histology.

## Data Availability

The data presented in this study are openly available in the Surveillance, Epidemiology, and End Results database.

## References

[1] Zavala V.A., Bracci P.M., Carethers J.M., Carvajal-Carmona L., Coggins N.B., Cruz-Correa M.R., Davis M., de Smith A.J., Dutil J., Figueiredo J.C. (2021). Cancer health disparities in racial/ethnic minorities in the United States. Br. J. Cancer.

[2] Siegel R.L., Fedewa S.A., Anderson W.F., Miller K.D., Ma J., Rosenberg P.S., Jemal A. (2017). Colorectal Cancer Incidence Patterns in the United States, 1974–2013. J. Natl. Cancer Inst..

[3] Christopher E.S.N., Kazzi B., Lapen K., Franco I., Jain B., Patel Tej A., Mahal B., Rimner A., Wu A., Iyengar P. (2024). Disparities in Stage at Presentation Among Hispanic and Latinx Patients With Non–Small-Cell Lung Cancer in the United States. JCO Oncol. Pract..

[4] Chien C., Morimoto L.M., Tom J., Li C.I. (2005). Differences in colorectal carcinoma stage and survival by race and ethnicity. Cancer.

[5] Menashe I., Anderson W.F., Jatoi I., Rosenberg P.S. (2009). Underlying causes of the black-white racial disparity in breast cancer mortality: A population-based analysis. J. Natl. Cancer Inst..

[6] Gupta D.R., Liu Y., Jiang R., Walid S., Higgins K., Landry J., McDonald M., Willingham F.F., El-Rayes B.F., Saba N.F. (2019). Racial Disparities, Outcomes, and Surgical Utilization among Hispanics with Esophageal Cancer: A Surveillance, Epidemiology, and End Results Program Database Analysis. Oncology.

[7] U.S. Census Bureau QuickFacts: United States [Internet]. United States Census Bureau; 2023. https://www.census.gov/quickfacts/fact/table/US/RHI725222.

[8] Siegel R.L., Miller K.D., Wagle N.S., Jemal A. (2023). Cancer statistics, 2023. CA Cancer J. Clin..

[9] Long Parma D., Schmidt S., Munoz E., Ramirez A.G. (2021). Gastric adenocarcinoma burden and late-stage diagnosis in Latino and non-Latino populations in the United States and Texas, during 2004–2016: A multilevel analysis. Cancer Med..

[10] Collaborators GUHD (2023). The burden of stomach cancer mortality by county, race, and ethnicity in the USA, 2000–2019: A systematic analysis of health disparities. Lancet. Reg. Health Am..

[11] Surveillance, Epidemiology, and End Results (SEER) Program SEER*Stat Database: Incidence—SEER Research Data, 8 Reg-istries, Nov 2022 Sub (1975–2020). www.seer.cancer.gov.

[12] STATA 2023 [V17.0:]. https://www.stata.com.

[13] Canedo J.R., Miller S.T., Schlundt D., Fadden M.K., Sanderson M. (2018). Racial/Ethnic Disparities in Diabetes Quality of Care: The Role of Healthcare Access and Socioeconomic Status. J. Racial. Ethn. Health Disparities.

[14] Sentell T., Braun K.L. (2012). Low health literacy, limited English proficiency, and health status in Asians, Latinos, and other racial/ethnic groups in California. J. Health Commun..

[15] Wells K.B., Golding J.M., Hough R.L., Burnam M.A., Karno M. (1989). Acculturation and the probability of use of health services by Mexican Americans. Health Serv. Res..

[16] Perez-Escamilla R., Garcia J., Song D. (2010). Health care access among hispanic immigrants: Inverted question markalguien esta escuchando? [Is anybody listening?]. NAPA Bull..

[17] Mehta N., Raker C., Robison K. (2021). Cervical Cancer Prevention: Screening among Undocumented Hispanic Women Compared with Documented Hispanic Women. J. Low Genit. Tract. Dis..

[18] Echeverria S.E., Carrasquillo O. (2006). The roles of citizenship status, acculturation, and health insurance in breast and cervical cancer screening among immigrant women. Med. Care.

[19] Vitiello G.A., Hani L., Wang A., Porembka M.R., Alterio R., Ju M., Turgeon M.K., Lee R.M., Russell M.C., Kronenfeld J. (2021). Clinical Presentation Patterns and Survival Outcomes of Hispanic Patients with Gastric Cancer. J. Surg. Res..

[20] Diamond L., Izquierdo K., Canfield D., Matsoukas K., Gany F. (2019). A Systematic Review of the Impact of Patient-Physician Non-English Language Concordance on Quality of Care and Outcomes. J. Gen. Intern. Med..

[21] Parker M.M., Fernandez A., Moffet H.H., Grant R.W., Torreblanca A., Karter A.J. (2017). Association of Patient-Physician Language Concordance and Glycemic Control for Limited-English Proficiency Latinos with Type 2 Diabetes. JAMA Intern. Med..

[22] Torrejon N.D.S., Wei W., Tullio K., Kamath S. (2022). Proportion of Early-Onset Gastric and Esophagus Cancers Has Changed Over Time with Disproportionate Impact on Black and Hispanic Patients. JCO Oncol. Pract..

[23] Ma Z., Liu X., Paul M.E., Chen M., Zheng P., Chen H. (2021). Comparative investigation of early-onset gastric cancer. Oncol. Lett..

[24] Warner D., Procaccino J.D. (2007). Women seeking health information: Distinguishing the web user. J. Health Commun..

[25] Manierre M.J. (2015). Gaps in knowledge: Tracking and explaining gender differences in health information seeking. Soc. Sci. Med..

[26] Galdas P.M., Cheater F., Marshall P. (2005). Men and health help-seeking behaviour: Literature review. J. Adv. Nurs..

[27] Toal T.W., Estrada-Florez A.P., Polanco-Echeverry G.M., Sahasrabudhe R.M., Lott P.C., Suarez-Olaya J.J., Guevara-Tique A.A., Rocha S., Morales-Arana A., Castro-Valencia F. (2022). Multiregional Sequencing Analysis Reveals Extensive Genetic Heterogeneity in Gastric Tumors from Latinos. Cancer Res. Commun..

[28] Zhang G., Zhao X., Li J., Yuan Y., Wen M., Hao X., Li P., Zhang A. (2017). Racial disparities in stage-specific gastric cancer: Analysis of results from the Surveillance Epidemiology and End Results (SEER) program database. J. Investig. Med..

[29] Williams M.H., Williams R.A., Hernandez B., Michalek J., Parma D.L., Arora S.P. (2021). Clinicopathologic differences and mortality among Latinos and non-Latino whites with gastric cancer at a majority-minority cancer center in South Texas. J. Gastrointest. Oncol..

[30] Jin H., Pinheiro P.S., Callahan K.E., Altekruse S.F. (2017). Examining the gastric cancer survival gap between Asians and whites in the United States. Gastric. Cancer.

[31] Cook E.D., Yeager K.A., Cecchini R.S., Boparai J., Brown C.L., Duncan M., Cronin W.M., Paskett E.D. (2018). Recruitment practices for U.S. minority and underserved populations in NRG oncology: Results of an online survey. Contemp. Clin. Trials Commun..

[32] Duma N., Vera Aguilera J., Paludo J., Haddox C.L., Gonzalez Velez M., Wang Y., Leventakos K., Hubbard J.M., Mansfield A.S., Go R.S. (2018). Representation of Minorities and Women in Oncology Clinical Trials: Review of the Past 14 Years. J. Oncol. Pract..

[33] Liu D., Schuchard H., Burston B., Yamashita T., Albert S. (2021). Interventions to Reduce Healthcare Disparities in Cancer Screening Among Minority Adults: A Systematic Review. J. Racial Ethn. Health Disparities.

